# Impact of introducing multiple evidence-based clinical practice protocols in a medical intensive care unit: a retrospective cohort study

**DOI:** 10.1186/1471-227X-7-10

**Published:** 2007-08-08

**Authors:** Bekele Afessa, Ognjen Gajic, Mark T Keegan, Edward G Seferian, Rolf D Hubmayr, Steve G Peters

**Affiliations:** 1 Division of Pulmonary and Critical Care Medicine, Department of Internal Medicine, Mayo Clinic College of Medicine, Rochester, Minnesota, USA; 2 Division of Critical Care, Department of Anesthesia, Mayo Clinic College of Medicine, Rochester, Minnesota, USA; 3 Division of Pediatric Critical Care Medicine, Department of Pediatric and Adolescent Medicine, Mayo Clinic College of Medicine, Rochester, Minnesota, USA

## Abstract

**Background:**

Recently completed clinical trials have shown that certain interventions improve the outcome of the critically ill. To facilitate the implementation of these interventions, professional organizations have developed guidelines. Although the impacts of the individual evidence-based interventions have been well described, the overall impact on outcome of introducing multiple evidence-based protocols has not been well studied. The objective of this study was to determine the impact of introducing multiple evidence-based protocols on patient outcome.

**Methods:**

A retrospective, cohort study of 8,386 patients admitted to the medical intensive care unit (MICU) of an academic, tertiary medical center, from January 2000 through June 2005 was performed. Four evidence-based protocols (lung protective strategy for acute lung injury, activated protein C for severe sepsis/septic shock, intravenous insulin for hyperglycemia control and a protocol for sedation/analgesia) were introduced in the MICU between February 2002 and April 2004. We considered the time from January 2000 through January 2002 as the pre-protocol period, from February 2002 through March 2004 as the transition period and from April 2004 through June 2005 as the protocol period. We retrieved data including demographics, severity of illness as measured by the Acute Physiology and Chronic Health Evaluation (APACHE) III, MICU length of stay and hospital mortality. Student's t, Kruskal-Wallis, Mann-Whitney U, chi square and multiple logistic regression analyses were used to compare differences between groups. P-values < 0.05 were considered significant.

**Results:**

The predicted mean mortality rates were 20.7%, 21.1% and 21.8%, with the observed mortality rates of 19.3%, 18.0% and 16.9% during the pre-protocol, transition and protocol periods, respectively. Using the pre-protocol period as a reference, the severity-adjusted risk (95% confidence interval) of dying was 0.777 (0.655 – 0.922) during the protocol period (P = 0.0038). The average 28-day MICU free days improved during the protocol period compared to the pre-protocol period. The benefit was limited to sicker patients and those who stayed in the MICU longer.

**Conclusion:**

The introduction of multiple evidence-based protocols is associated with improved outcome in critically ill medical patients.

## Background

Although the numbers of acute care hospitals and hospital beds have declined over the last two decades, the number of intensive care unit (ICU) beds has risen [1]. Despite the proliferation of intensive care units, there had not been compelling evidence to guide critical care practice until recent randomized clinical trials showed that certain clinical practices improve the outcome of the critically ill. The daily interruption of intravenous sedative medications in patients receiving invasive mechanical ventilation reduces the length of ICU stay and the duration of mechanical ventilation [2]. Early goal-directed therapy for severe sepsis and septic shock [3], lung protective strategy for acute lung injury (ALI) [4], control of hyperglycemia using intravenous insulin [5,6] and the use activated protein C for severe sepsis [7] reduce the morbidity and mortality of critically ill patients.

Although there is ample evidence supporting the benefit that certain interventions improve the clinical outcome of the critically ill, including length of stay, duration of mechanical ventilation, and mortality, there are barriers to translating the evidence into clinical practice [8-10]. To overcome these barriers, professional organizations [11,12] and individual medical centers [13-16] have developed international and local guidelines and protocols based on the available evidence. Federal and state governments, insurers and accreditation bodies have also reached a consensus recognizing the importance of evidence based practice and quality measurement [17]. Currently, the Centers for Medicare and Medicaid Services are developing and implementing a set of pay-for-performance initiatives to support quality improvement in the care of Medicare and Medicaid beneficiaries in the Unites States of America [18].

Although the impacts of the individual evidence-based interventions have been well described, the overall impact on outcome of introducing multiple evidence-based protocols has not been well studied. We have implemented multiple, evidence-based protocols in our medical intensive care unit (MICU) in the last 4 years. We have an Acute Physiology and Chronic Health Evaluation (APACHE) III database that is used for severity adjusted outcome measure including mortality and length of stay [19]. We introduced four evidence-based clinical protocols (lung protective strategy for ALI, activated protein C for septic shock, intravenous insulin for hyperglycemia control, and sedation/analgesia protocol) in the MICU between February 2002 and April 2004. This study assesses the impact of introducing these evidence-based protocols on patient outcome based on our APACHE III database.

## Methods

In this retrospective, cohort study, we reviewed the APACHE III database of patients admitted to the MICU of Mayo Medical Center, Rochester, Minnesota. Mayo Medical Center is a tertiary, teaching institution with two hospitals comprising approximately 1,900 in-patient beds. The study was approved by the Mayo Foundation Institutional Review Board. The MICU was a closed unit throughout the study period. It had a 15-bed capacity at the beginning of the study period. The capacity was increased to 19-bed in August 2002 and 24-bed in December 2002. A critical care service team consisting of attending intensivists, critical care fellows, residents, pharmacists, nurses and respiratory therapists staffed the MICU. The non-physician staffing was consistent throughout the study period. The nurse to patient ratio was 1 to 1 or 1 to 2. Nurses, pharmacists and respiratory therapists participated during the daily rounds. All attending intensivists had internal medicine background and critical care or pulmonary/critical care subspecialty training. Fellows and internal medicine residents provided 24-hour in-house coverage. The attending intensivists did not routinely stay in-house at night but were available by phone and came to the ICU as needed. Clinically important decisions in the ICU were made by, or under the direct supervision of, the attending intensivists.

Patients who did not authorize their medical records to be reviewed for research were excluded. Data retrieved included demographics, MICU admission diagnosis; Acute Physiology Score (APS), APACHE III score, and hospital predicted mortality rate based on the first MICU day values; length of MICU stay and hospital mortality. The hospital predicted mortality rates were calculated based on the admission diagnoses, APACHE III score and location prior to MICU admission, using software provided by Cerner Corporation (Kansas City, Missouri) [20]. Subgroup analyses were performed based on the severity of illness and the length of MICU stay. The severity of illness was categorized into high and low using the median predicted hospital death as a cutoff point. The MICU length of stay was categorized into long and short using the median MICU length of stay as a cutoff point.

We developed and started implementing four evidence-based protocols as follows: lung protective strategy for ALI in February 2002, activated protein C for severe sepsis/septic shock in October 2002, intravenous insulin for hyperglycemia control in September 2003 and a protocol for sedation/analgesia in April 2004. The protocols were developed with the participation of all MICU staff including physicians, nurses, respiratory therapists and pharmacists. The protocol for lung protective strategy was based on providing tidal volume not greater than 6 mL/kg ideal body weight in patients with ALI or Acute Respiratory Distress syndrome (ARDS). The activated protein C protocol was applicable for adults with severe sepsis/septic shock and multiple organ failure with no risk factor for bleeding and who opted for full resuscitation and life support. The hyperglycemia control protocol was activated if patients' glucose was > 150 mg/dL. A continuous intravenous insulin infusion was titrated to maintain blood glucose level between 100 and 119 mg/dL. There was also a protocol for the treatment of hypoglycemia based on symptoms or blood glucose level < 60 mg/dL. The sedation/analgesia protocol had two parts, one for patients anticipated to remain intubated for 48 hours or less and another protocol for those expected to remain intubated longer than 48 hours. The less than 48 hours sedations/analgesia protocol used morphine or fentanyl for analgesia and propofol or midazolam for sedation. The longer than 48-hour protocol used morphine or fentanyl for analgesia and lorazepam for sedation. Both analgesia/sedation protocols used numeric pain scales and Richmond Agitation-Sedation Scale (RASS) for titration and the continuous infusion of the opioids and sedatives was interrupted daily. (The sedation/analgesia protocols are available as additional file 1 and 2.)

The development of each protocol had taken several months before implementation. Once implemented, no significant modifications were made in any one of the protocols during the study period. We considered the times from January 2000 through January 2002 as the pre-protocol period, from February 2002 through March 2004 as the transition period (since introduction of the three protocols started during this period) and from April 2004 through June 2005 as the protocol period (all four protocols were implemented). During the pre-protocol period, the implementation of evidence-based practice was based on the individual physicians' knowledge and discretion. During the development of the protocols, all MICU staff became more familiar with the available evidence. With the activation of the protocols, the elements that constitute evidence-based practice were easily available in order-set forms and were implemented with the active participation of intensivists, fellows, residents, nurses, respiratory therapists and pharmacists.

The 28-day ICU free days were calculated by subtracting the actual ICU length of stay in days from 28. The 28-day ICU free day was considered 0 if a patient died before hospital discharge or stayed in the ICU for > 28 days [21,22]. We used the 28-day ICU free days to avoid the confounding effect of mortality. This number measured the time interval that a patient was both alive and did not require ICU support.

We summarized data as mean (standard deviation) (SD), median (interquartile range) (IQR) or percentages. Student's t, Kruskal-Wallis, Mann-Whitney U, and chi square tests were used to compare differences between groups. In order to determine the impact of the protocol on severity adjusted patient outcome, we created a multiple logistic regression model consisting of hospital mortality as a dependent variable and the APACHE III predicted hospital mortality rate and the three study periods as independent variables. All independent variables were entered into the model simultaneously. The pre-protocol period was considered as reference in this logistic regression model. When appropriate, the 95% confidence intervals (CI) were calculated. We considered P values < 0.05 as statistically significant. We used StatView version 5.0 (SAS Inc, Cary, North Carolina) and Confidence Interval Analysis version 2.0.5 (Trevor Bryant, University of Southampton, United Kingdom) computer softwares for statistical analysis. We used variable life-adjusted display (VLAD) to show the differences between the cumulative and actual deaths during the three periods of the study [23,24].

## Results

After exclusion of 186 patients (2.2%) who did not authorize their medical records to be reviewed for research, 8,386 patients were included in the study. Of the 8,386 patients, 7,910 (94.3%) were white and 4,346 (51.8%) were male. Their mean (SD) age was 62.3 (19.1) years. The mean APS, APACHE III score and predicted hospital mortality rates were 46.5, 60.1 and 21.1%, respectively. The number of patients included in the study was 2,677 in the pre-protocol, 3,513 in the transition and 2,196 in the protocol periods (Table 1).

**Table 1:** Difference in base-line characteristics among the three study periods*

Characteristics	Pre-protocol N = 2,677	Transition N = 3,513	Protocol N = 2,196
Age, yrs, mean ± SD	61.7 ± 19.3	62.3 ± 19.0	63.0 ± 19.1†
Male sex	1,405 (52.5)	1,785 (50.8)	1,156 (52.6)
White race	2,526 (94.4)	3,306 (94.1)	2,078 (94.6)
APS, Median (IQR)	40 (25–59)	41 (27–59)†	43 (30–60)†
APACHE III, Median (IQR)	55 (37–76)	56 (39–75)	58 (42–77)†
Predicted mortality, %, Median (IQR)	11.3 (3.1–29.7)	11.5 (3.5–29.2)	13.0 (4.4–31.0)†

APS = Acute Physiology Score; APACHE = Acute Physiology and Chronic Health Evaluation; IQR = Interquartile range

*Values are number (percentage) unless indicated otherwise; †P < 0.05 compared to pre-protocol period.

**Figure 1:**
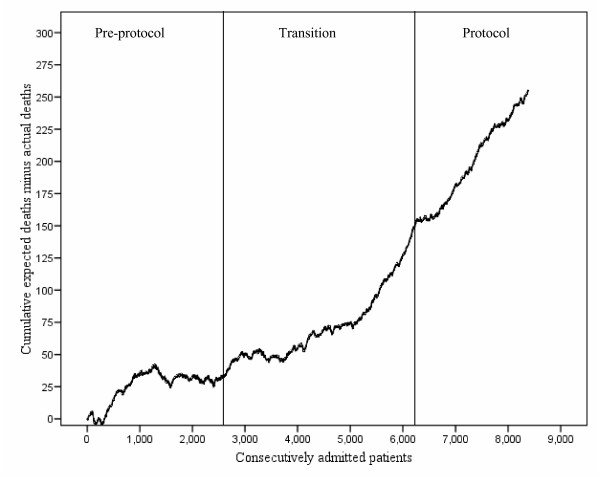
Variable life-adjusted display (VLAD) for 8,386 patients consecutively admitted to the medical intensive care unit during the three study periods (Pre-protocol, Transition, and Protocol).

The protocol patients were older than the pre-protocol patients (Table 1). Although the differences were small, the APS, APACHE III score and predicted hospital mortality rate were higher in the protocol period compared to the pre-protocol period (Table 1). Compared to the pre-protocol group, the 28-day ICU free days were longer in the protocol group (Table 2). Although the actual reduction was small, the logistic regression model showed that the severity adjusted hospital mortality rate was significantly reduced during the protocol period compared to the pre-protocol period (Table 3). The VLAD shows an overall improvement in mortality during the protocol period (Figure 1).

**Table 2:** Difference in length of stay and hospital mortality among the three study periods

Outcome	Pre-protocol N = 2,677	Transition N = 3,513	Protocol 2,196
Observed mortality, %, (95% CI)	19.3 (17.8 – 20.8)	18.0 (16.7 – 19.3)	16.9 (15.4 – 18.5)
ICU length of stay, days, median (IQR)	1.6 (0.85–3.29)	1.4 (0.84–2.98) *	1.7 (0.91–3.14)
28-day ICU free days, days, mean ± SD	20.4 ± 10.6	20.8 ± 10.3	21.1 ± 10.0*

CI = confidence interval; ICU = intensive care unit; IQR = interquartile range; SD = standard deviation

* P < 0.05 compared to pre-protocol period

**Table 3:** The association of the protocol periods with hospital mortality adjusted for the severity of illness

Variable	Odds ratio (95% CI)	P-value
Predicted mortality, %	1.049* (1.046 – 1.052)	< 0.001
Study period		
Pre-protocol	Reference	
Transition	0.869 (0.747–1.010)	0.067
Protocol	0.777 (0.655–0.922)	0.004

CI = Confidence interval

*This odds ratio reflects the risk of death per 1% rise in the predicted mortality rate for the entire study population.

The median predicted hospital death rate and ICU length of stay were 11.8% and 1.6 days, respectively. The mortality benefit of the protocol period was limited to the high severity and longer MICU stay groups (Table 4). There were small differences in the 28-day ICU free days favoring the protocol period compared to the pre-protocol period in patients with higher severity of illness (Table 5).

**Table 4:** Subgroup analyses describing the association of the protocol periods with severity-adjusted hospital mortality

Subgroup	Variable	Odds ratio (95% CI)	P-value
High severity of illness			
	Predicted mortality, %	1.040* (1.036 – 1.043)	< 0.001
	Protocol period		
	Pre-protocol	Reference	
	Transition	0.831 (0.701–0.984)	0.031
	Protocol	0.754 (0.625–0.910)	0.003
Low severity of illness			
	Predicted mortality, %	1.241* (1.191 – 1.293)	< 0.001
	Protocol period		
	Pre-protocol	Reference	
	Transition	1.013 (0.730–1.406)	0.938
	Protocol	0.733 (0.492–1.093	0.128
Long ICU length of stay			
	Predicted mortality, %	1.033* (1.029 – 1.036)	< 0.001
	Protocol period		
	Pre-protocol	Reference	
	Transition	0.893 (0.746–1.069)	0.217
	Protocol	0.745 (0.609–0.910)	0.004
Short ICU length of stay			
	Predicted mortality, %	1.077* (1.071 – 1.083)	< 0.001
	Protocol period		
	Pre-protocol	Reference	
	Transition	0.829 (0.620–1.109)	0.207
	Protocol	0.953 (0.686–1.325)	0.776

CI = Confidence interval

*This odds ratio reflects the risk of death per 1% rise in the predicted mortality rate for the entire study population.

**Table 5:** Difference in length of intensive care unit stay between the three study periods in severity subgroups of the study population

Subgroup and outcome	Pre-protocol	Transition	Protocol
High severity of illness			
ICU length of stay, days, median (IQR)	2.4 (1.13–5.02)	2.06 (1.05–4.64)*	2.4 (1.21–4.71)
28–day ICU free days, median (IQR)	23.2 (0.0–26.2)	24.1 (0.0–26.4) *	24.4 (0.0–26.3)*
Low severity of illness			
ICU length of stay, days, median (IQR)	1.0 (0.75–1.99)	1.0 (0.72–1.93)	1.1 (0.77–1.99)
28-day ICU free days, median (IQR)	26.9 (25.9–27.3)	26.9 (26.0–27.3)	26.9 (25.9–27.2)

*P value < 0.05 compared to the pre-protocol period; ICU = intensive care unit; IQR = interquartile range; SD = standard deviation

## Discussion

In this study, we found that the introduction of multiple evidence-based clinical practice protocols was associated with a decline in severity-adjusted hospital mortality. We also noted that the 28-day ICU free days improved slightly. The benefits were limited to sicker patients and those who had longer ICU stay. The study suggests that the application of multiple evidence-based clinical practice protocols improves the clinical outcome of the critically ill.

Because of the complexity of intensive care units, the Institute for Healthcare Improvement (IHI) advocates use of protocol-based bundles in order to apply the best available science into clinical practice and improve patient outcome [25]. In the current study, the introduction of multiple evidence-based protocols was associated with reducing the severity-adjusted risk of hospital death. There are only few studies that addressed the impact of the application of multiple protocols on the outcome of critically ill patients. Previous studies have shown that the implementation of a ventilator bundle protocol (composed of stress ulcer prophylaxis, deep vein thrombosis prophylaxis, daily cessation of sedation and elevating the patient's head at least 30 degrees above the horizontal with or without daily assessment of readiness to wean from mechanical ventilation) reduces the ICU length of stay and duration of mechanical ventilation [13,26]. The IHI has initiated the various phases of Saving Lives Campaign. The campaign focuses on reducing mortality by implementing evidence-based practices and reducing errors. The findings in this study highlight the fact that implementation of evidence-based clinical practice protocols may help to achieve the objectives of the Saving Lives Campaign.

In the Acute Respiratory Distress Syndrome Network study, lung protective strategy reduced the mortality of patients with ALI from 39.8% to 31.0% [4]. van den Berghe and colleagues showed that intensive insulin therapy reduced mortality of a predominantly surgical critically ill patient population from 10.9% to 7.2% [5]. In a recent study of medical ICU patients by the same group, the survival benefit of intensive insulin therapy was limited to patients who stay in the ICU for more than three days [6]. In the overall MICU patient population, the hospital mortality rate associated with intensive insulin therapy (37.3%) was not statistically different from that of conventional treatment (40.0%). However, in patients who stayed in the medical ICU for more than three days, intensive insulin therapy was associated with reducing the hospital mortality rate from 52.5% to 43.0% [6]. In patients with severe sepsis, recombinant human activated protein C reduced the mortality rate from 30.8% to 24.7% [7]. The reduction in mortality observed in our study is consistent with the findings from the randomized clinical trials. With regard to recombinant human activated protein C, recent observations suggest that only a minority of eligible patients receive the treatment and it may have a detrimental effect in certain subgroups of patients [27,28].

The implications of reducing ICU days include reducing ICU complications and associated costs. The study by Kress and colleagues had shown reduction of ICU stay by 3.5 days using a protocol with daily interruption of sedative infusions [2]. The 28-day ICU free days were longer in the protocol period in our study by 0.7 days. With the shortage of staffed ICU beds in many medical centers, reducing the ICU length of stay has important implications, by decreasing the associated cost and avoiding delays in the care of patients waiting for ICU beds. Although we did not implement the sedation/analgesia protocol before 2004, it had been applied at the individual clinician's discretion, partly explaining why we did not see the dramatic effect reported by Kress et al. In our ICU, the critical care team made rounds at least twice daily. Even before the protocols were implemented, the critical care consultants who guided daily care and the fellows were aware of the studies that led to the protocols. In a study by Krishnan et al from Johns Hopkins medical institute, protocol-directed weaning did not improve patient outcome, including the ICU length of stay, compared to the usual care in a closed, generously staffed medical intensive care unit [29]. When the usual care is already influenced by the available evidence and in intensive care units where there is adequate physician staffing with daily structured multi-disciplinary rounds, the benefits of the protocols may not be as pronounced as in the original studies.

Our study has several limitations. Since the study was performed in a single medical center with its own unique characteristics, the findings may not be generalizable. The APACHE III database we used for the study did not include information on the rate of compliance with the protocols or evidence-based practice. We did not have the data to determine the eligibility and contraindications for each protocol. Our data also lacked the identification of the individual patients who received treatment based on the protocols. Since the four protocols were introduced at various times of the study, it is not easy to determine the effects of each protocol individually. We may not have accounted for all confounding variables although we adjusted for the severity of illness. Because of the retrospective design, our study cannot exclude the fact that unmeasured changes in patient care and unrelated to the protocols may have contributed to the improved outcome. The current study was performed over a period of 66 months. The transition period to having all the protocols available in the MICU took 24 months. Because of the long time interval it took to complete the study, we cannot avoid the potential confounding effects of frequently imperceptible changes in practice on outcome measures. Our study did not control for factors such as ICU and hospital patient volume and occupancy that may influence outcome. In the early part of our study, there were changes in the structure and staffing of the MICU. The MICU had expanded from 15 to 19 and then to 24 beds and the intensivist to bed ratio had changed from 1:15 to 1:9.5 and then to 1:15 [30]. However, we had shown these staffing and structural changes did not have significant impact on mortality in a previous publication [30]. Clinical researchers in our institution have monitored practice patterns and published studies focusing on ICU outcome in recent years. These reports may have had a Hawthorne effect. The large number of patients in our study may have led to statistically significant p values even when the clinical differences are of limited clinical value. For example, the difference in the 28-day ICU free days between the pre-protocol and protocol period was only 0.7 although the P value was < 0.05.

## Conclusion

The current study suggests that the introduction of several evidence-based patient care protocols is associated with improved (small albeit significant) severity adjusted mortality in a population of critically ill adult patients admitted to a medical ICU. Using sensitivity analysis, Pronovost and colleagues have extrapolated that 167,819 lives can be saved annually by the consistent and appropriate implementation of evidence-based therapies in the intensive care unit [31]. However, previous publications have highlighted the delay and reluctance in translating research findings into practice [8-10]. Errors of omission should not be tolerated. Future studies should address the barriers to the implementation of evidence-based clinical practices in the ICU and the potential solutions to the barriers.

## Abbreviations

ALI = Acute lung injury APACHE = Acute Physiology and Chronic Health Evaluation

APS = Acute Physiology Score

ARDS = Acute Respiratory Distress syndrome

CI = Confidence intervals

ICU = Intensive care unit

IQR = Interquartile range

MICU = Medical intensive care unit

RASS = Richmond Agitation-Sedation Scale

SD = Standard deviation

## Competing interests

The author(s) declare that they have no competing interests.

## Authors' contributions

BA was involved in the conception and design of the study, acquisition, analyzing and interpretation of data, writing of manuscript. OG was involved in the conception and design of the study, acquisition and interpretation of data, critical revision of the manuscript. MTK was involved in the conception and design of the study, interpretation of data, critical revision of the manuscript. EGS was involved in the conception and design of the study, interpretation of data, critical revision of the manuscript. RDH was involved in the conception and design of the study, interpretation of data, critical revision of the manuscript. SGP was involved in the conception and design of the study, interpretation of data, critical revision of the manuscript. All authors read and approved the final manuscript.

## Pre-publication history

The pre-publication history for this paper can be accessed here:



## Supplementary Material

Additional file 1 Sedation-analgesia protocol A. Describes the management of pain and agitation in patients anticipated to receive invasive mechanical ventilation for 48 hours or shorter Click here for file

Additional file 2 Sedation-analgesia protocol B. Describes the management of pain and agitation in patients anticipated to receive invasive mechanical ventilation for longer than 48 hours Click here for file
