# Supplementary material for: Impact of introducing multiple evidence-based clinical practice protocols in a medical intensive care unit: a retrospective cohort study
Source: BMC Emerg Med. 2007 Aug 8;7:10. doi: 10.1186/1471-227X-7-10 (PMC1965465; doi:10.1186/1471-227X-7-10)
Supplement: Additional file 1 — Sedation-analgesia protocol A. Describes the management of pain and agitation in patients anticipated to receive invasive mechanical ventilation for 48 hours or shorter [file 1471-227X-7-10-S1.pdf]

MAYO CLINIC

Management of Pain and Agitation: Mechanical Ventilation Anticipated For Less Than or Equal to 48 Hours

Orders apply to adult patients in intensive care units (15 years of age and older). Mark the ☒ for desired orders. If ☐ are blank, order is inactive. All pre-printed doses are based on normal renal and hepatic function and must be assessed for adjustment against the individual patient's renal and hepatic function and for interactions with other medications.

Name

Clinic Number

Room Number

Height: ________ cm Weight: ________ kg Age: ______

Allergy module reviewed. ☐ No additional allergies identified, **or**

Choose one: ☐ Additional allergies identified and MICS Allergy updated, **or**

☐ Additional allergies. List (include reaction): ________________ please update MICS Allergies.

ALERT

This order set does not apply to patients receiving neuromuscular blocking agents nor to patients receiving noninvasive mechanical ventilation.

In patients with endotracheal intubation and mechanical ventilation anticipated for greater than 48 hours, consider using order set MC1156-406 "ICU Management of Pain and Agitation in Patients with Endotracheal Intubation and Mechanical Ventilation Anticipated for Greater than 48 Hours".

MEDICATIONS:

1. Pain: Choose A or B.
Target pain rating less than 4.

A. Mean Arterial Pressure greater than 70 mmHg.
Morphine (Duramorph®) IV

☐ **1.** If pain greater than or equal to 4, give loading dose of ______ mg (2 – 5 mg). Repeat dose of ______ mg (2 – 5 mg) every 5 minutes for pain greater than or equal to 4 (maximum 30 mg in any one hour).

2. If pain greater than or equal to 4 after 5 total doses in any one hour, begin continuous infusion at ______ mg (2 – 10 mg) per hour.

Repeat dose of ______ mg (2 – 5 mg) every 5 minutes for pain greater than or equal to 4 (maximum 30 mg in any one hour).

3. If pain greater than or equal to 4 after 5 total doses in any one hour after continuous infusion, increase continuous infusion by 1 mg per hour every hour until pain rating less than 4 (maximum 15 mg per hour).

☐ Other ________________

B. Mean Arterial Pressure less than 70 mmHg, or if allergic to Morphine (Duramorph®):
Fentanyl (Sublimaze®) IV

☐ **1.** If pain greater than or equal to 4, give loading dose of ______ mcg (25 – 50 mcg). Repeat dose of ______ mcg (25 – 50 mcg) every 5 minutes for pain greater than or equal to 4 (maximum 600 mcg in any one hour).

2. If pain greater than or equal to 4 after 5 total doses in any one hour, begin continuous infusion at ______ mcg (25 – 100 mcg) per hour.

Repeat dose of ______ mcg (25 – 50 mcg) every 5 minutes for pain greater than or equal to 4 (maximum 600 mcg in any one hour).

3. If pain greater than or equal to 4 after 5 total doses in any one hour after continuous infusion, increase continuous infusion by 25 mcg per hour every hour until pain rating less than 4 (maximum 300 mcg per hour).

☐ Other ________________

2. Agitation (if pain rating less than 4): Choose A or B.
Target sedation to 0 on Richmond Agitation Sedation Scale (RASS); see the reverse of sheet.

A. Mean Arterial Pressure greater than 70 mmHg (choose a or b):

a. Propofol (Diprivan®) IV

☐ **1.** If RASS score greater than or equal to +1, give loading dose of ______ mg (0.3 mg/kg) and begin continuous infusion at ______ mcg/kg (5 – 80 mcg/kg) per minute.

Increase by 5 mcg/kg per minute until RASS score equal to 0 (maximum 80 mcg/kg per minute).

☐ Other ________________

b. Midazolam (Versed®) IV

☐ **1.** If RASS score greater than or equal to +1, give loading dose of ______ mg (1 – 4 mg). Repeat dose of ______ mg (1 – 4 mg) every 5 minutes if RASS score greater than or equal to +1 (maximum 40 mg in any one hour).

2. If RASS score greater than or equal to +1 after 5 total doses in any one hour, begin continuous infusion at ______ mg (1 – 4 mg) per hour.

Repeat dose of ______ mg (1 – 4 mg) every 5 minutes if RASS score greater than or equal to +1 (maximum 40 mg in any one hour).

3. If RASS score greater than or equal to +1 after 5 total doses in any one hour after continuous infusion, increase continuous infusion by 1 mg per hour every hour until RASS score equal to 0 (maximum 20 mg per hour).

B. Mean Arterial Pressure less than 70 mmHg, or if allergic to Propofol (Diprivan®) or eggs:
Midazolam (Versed®) IV

☐ **1.** If RASS score greater than or equal to +1, give loading dose of ______ mg (1 – 4 mg). Repeat dose of ______ mg (1 – 4 mg) every 5 minutes if RASS score greater than or equal to +1 (maximum 40 mg in any one hour).

2. If RASS score greater than or equal to +1 after 5 total doses in any one hour, begin continuous infusion at ______ mg (1 – 4 mg) per hour.

Repeat dose of ______ mg (1 – 4 mg) every 5 minutes if RASS score greater than or equal to +1 (maximum 40 mg in any one hour).

3. If RASS score greater than or equal to +1 after 5 total doses in any one hour after continuous infusion, increase continuous infusion by 1 mg per hour every hour until RASS score equal to 0 (maximum 20 mg per hour).

☐ Other ________________

3. ☒ This order set is to be discontinued once the patient is extubated and no longer receiving mechanical ventilation.

Prescriber's Signature: ________________ Prescriber's Pager #: ________ Service Pager #: ________

Prescriber's Printed Name: ________________ Date: mm / dd / yyyy Time: hh : mm (24 hour clock)

Part 1 – Pharmacy Part 2 – Nursing Part 3 – Order Book

This order set has been developed to reflect the practice patterns of the clinicians who wrote it. It sets forth recommendations as to practice, not rigid rules.

© 2004 Mayo Foundation for Medical Education and Research. All rights reserved.

Richmond Agitation-Sedation Scale (RASS)

Score	Term	Description
+4	Combative	Overtly combative; violent; immediate danger to staff
+3	Very agitated	Pulls or removes tube(s) or catheter(s); aggressive
+2	Agitated	Frequent nonpurposeful movement, fights ventilator
+1	Restless	Anxious but movements not aggressive or vigorous
0	Alert and calm	
-1	Drowsy	Not fully alert, but has sustained awakening (Eye opening/eye contact) to voice (> 10 seconds)
-2	Light sedation	Briefly awakens with eye contact to voice (< 10 seconds)
-3	Moderate sedation	Movement or eye opening to voice (but no eye contact)
-4	Deep sedation	No response to voice, but movement or eye opening to physical stimulation
-5	Unarousable	No response to voice or physical stimulation

Procedure for RASS assessment

1. Observe patient
   - Patient is alert, restless, or agitated. Score 0 to +4

2. If not alert, state patient's name and say to open eyes and look at speaker.
   - Patient awakens with sustained eye opening and eye contact. Score –1
   - Patient awakens with eye opening and eye contact, but not sustained. Score –2
   - Patient has any movement in response to voice but no eye contact. Score –3

3. When no response to verbal stimulation, physically stimulate patient by Shaking shoulder and/or rubbing sternum.
   - Patient has any movement to physical stimulation. Score –4
   - Patient has no response to any stimulation. Score –5

Sessler, C. (2002). American Journal of Respiratory Critical Care Medicine. The Richmond Agitation-Sedation Scale: validity and reliability in adult intensive care unit patients. 166, 1338-1344.
